# Immunoinformatics approach for multi-epitope vaccine design against structural proteins and ORF1a polyprotein of severe acute respiratory syndrome coronavirus-2 (SARS-CoV-2)

**DOI:** 10.1186/s40794-021-00147-1

**Published:** 2021-07-08

**Authors:** Khalid Mohamed Adam

**Affiliations:** College of Applied Medical Sciences, Medical and Engineering Complex, University of Bisha, Bisha, 61922 Kingdom of Saudi Arabia

**Keywords:** Immunoinformatics, Multi-epitope, Vaccine, Coronavirus, COVID-19, SARS-COV-2

## Abstract

**Background:**

The lack of effective treatment against the highly infectious SARS-CoV-2 has aggravated the already catastrophic global health issue. Here, in an attempt to design an efficient vaccine, a thorough immunoinformatics approach was followed to predict the most suitable viral proteins epitopes for building that vaccine**.**

**Methods:**

The amino acid sequences of four structural proteins (S, M, N, E) along with one potentially antigenic accessory protein (ORF1a) of SARS-CoV-2 were inspected for the most appropriate epitopes to be used for building the vaccine construct. Several immunoinformatics tools were used to assess the antigenicity (VaxiJen server), immunogenicity (IEDB immunogenicity tool), allergenicity (AlgPred), toxigenicity (ToxinPred server), interferon-gamma inducing capacity (IFNepitope server), and the physicochemical properties of the construct (ProtParam tool).

**Results:**

The final candidate vaccine construct consisted of 468 amino acids, encompassing 29 epitopes. The CTL epitopes that passed the antigenicity, allergenicity, toxigenicity and immunogenicity assessment were four epitopes from S protein, one from M protein, two from N protein, 12 from the ORF1a polyprotein and none from E protein. While the HTL epitopes that passed the antigenicity, allergenicity, toxigenicity and INF-$\gamma$ were one from S protein, three from M protein, six from the ORF1a polyprotein and none from N and E proteins.

All the vaccine properties and its ability to trigger the humoral and cell-mediated immune response were validated computationally. Molecular modeling, docking to TLR3, simulation, and molecular dynamics were also carried out. Finally, a molecular clone using pET28::mAID expression plasmid vector was prepared.

**Conclusion:**

The overall results of the study suggest that the final multi-epitope chimeric construct is a potential candidate for an efficient protective vaccine against SARS-CoV-2.

## Introduction

In early December 2019, an acute respiratory disease of unknown etiology emerged in Wuhan, China, which was subsequently found to be caused by a novel coronavirus. The virus was initially described as 2019-nCoV and later named by the international committee on taxonomy of viruses (ICTV) as severe acute respiratory syndrome coronavirus-2 (SARS-CoV-2), while the World Health Organization (WHO) named the disease Coronavirus disease-19 (COVID-19) [1–5]. Within the first three months after its discovery, the disease spread to more than 100 countries and caused more than 4,000 deaths worldwide [6]. On the 11th of March, 2020, the WHO categorized the newly discovered disease as a pandemic. COVID-19 is characterized by a broad clinical spectrum, ranging from asymptomatic, to mild to severe respiratory illness requiring intubation and intensive care. The disease course and outcome are contingent on a number of factors, such as age and presence of underlying comorbidities [7]. The clinical manifestations include fever, fatigue, nonproductive cough, dyspnea and myalgia. In severe cases, acute respiratory distress syndrome (ARDS), acute cardiac injury, and acute kidney injury and death can also occur [8, 9].

SARS-CoV-2 along with severe acute respiratory syndrome coronavirus (SARS-CoV) and Middle East respiratory syndrome coronavirus (MERS-CoV) are Betacoronaviruses belonging to the subfamily *Othrocoronavirinae* of the *Nidovirales.* These are enveloped, non-segmented, single-stranded, positive-sense RNA viruses, with genomes ranging from 26 to 32 Kb. The genome size of SARS-CoV-2 varies from 29.8 to 29.9 Kb, with typical genome structure of earlier well-characterized coronaviruses, such as the overlapping open reading frame 1a (ORF1a) and 1ab (ORF1ab) region and genes encoding four structural proteins including spike proteins (S), envelope proteins (E), membrane proteins (M), and nucleocapsid proteins (N), in addition to accessory proteins coding genes ORF3a, ORF6, ORF7a, ORF7b and ORF8 [10–13]. The main role of the spike (S) glycoproteins is to mediate binding to the angiotensin-converting enzyme 2 (ACE-2) receptor and promote membrane fusion and virus entry [14]. Both M and E proteins were reported to play important roles in viral entry, replication, and virions assembly [15]. N proteins are important for viral RNA packaging, virions release and interferon inhibition, promoting the virus pathogenicity [16, 17]. In SARS-CoV, the gene for N protein is upregulated, producing large amounts of the highly immunogenic protein [18]. On the other hand, ORF1a encodes nonstructural polyproteins (PP1a), these polyproteins are involved in viral genome replication and transcription [19].

The COVID-19 pandemic has affected all walks of life, stretching health-care systems to their maximum and putting a huge economical, psychological, and mental burden on the entire world population. This dire situation is aggravated by the contagious nature of the virus, lack of complete understanding of the disease course and the absence of a reliable cure [6]. The disease containment measures used thus far, are contingent on disrupting the transmissibility of the virus through rapid identification and isolation of infected and carrier individuals. This entails the search for vaccines and effective treatments. Recently, number of newly developed vaccines were granted emergency use authorization in many countries worldwide, these are mRNA vaccines such as BNT162b2, and mRNA-1273, DNA vaccines such as AZD1222, Ad26.COV2.S and Sputnik V, inactivated virus vaccines such as CoronaVac and BBIBP-CorV, and protein subunit vaccines such as NVX-CoV2373. Most of these vaccines rely mainly on S protein epitopes, and showed very promising results during different trial phases but they are being closely monitored for any issues regarding their effectiveness and safety. The aim of this study is to design a multi-epitope vaccine against SARS-CoV-2 based on four structural proteins along with the nonstructural polyprotein of ORF1a, using an immunoinformatics approach.

The selection of the nonstructural ORF1a polyprotein alongside the structural viral proteins in this study was driven by suggestions made by a number of studies on other viruses, that nonstructural polyproteins induce immunity and may be applicable to prophylaxis of viral disease [20–23]. ORF1a was selected over the larger ORF1ab because these two regions overlap, and most of the important proteins found in the region are covered by ORF1a. In addition, ORF1ab is the largest region in viral genome with possibility of larger number of potential epitopes, which in turn may increase the size of the construct to the point that the molecular weight of the final vaccine product will be too large and hinders its effectiveness and delivery.

## Materials and methods

### Retrieval of target proteins sequences

The amino acid sequences for S protein of 1273 amino acid (Accession No. QLI51913.1), M protein of 222 amino acid (Accession No. QLI52072.1), E protein of 75 amino acid (Accession No. QLI52071.1), N protein of 419 amino acid (Accession No. QIH45060.1) and ORF1a polyprotein of 4405 amino acid (Accession No. QJQ84087.1) were retrieved from NCBI protein database (https://www.ncbi.nlm.nih.gov/protein) in FASTA format.

### Cytotoxic T- cell lymphocyte (CTL) epitopes prediction

Initially, the amino acid sequences of all 5 proteins were screened for antigenicity with VaxiJen 2.0 server (http://www.ddg-pharmfac.net/vaxijen/VaxiJen/VaxiJen.html), with threshold value of 0.4 [24]. The CTL epitopes for all sequences were predicted using artificial neural network algorithm-based NetCTL 1.2 server (http://www.cbs.dtu.dk/services/NetCTL/), with threshold value of 0.75 which indicates 0.80 sensitivity and 0.97 specificity [25], which predicts major histocompatibility complex-1 (MHC-1) binding epitopes. The peptides obtained were then checked for antigenicity using VaxiJen 2.0 server. The antigenic peptides were then submitted for virtual scanning for toxic peptides using ToxinPred server (http://crdd.osdd.net/raghava/toxinpred/multi_submit.php), with threshold value 0.0 [26]. The immunogenicity of the resultant non-toxin epitopes was determined using class I immunogenicity tool of Immune Epitope Database (IEDB) (http://tools.iedb.org/immunogenicity/), version 2.22 [27].

### Helper T-lymphocyte (HTL) epitopes prediction

For prediction of HTL epitopes, MHC-II binding tool of IEDB (http://tools.iedb.org/mhcii/) was used [28], selecting 7-allele HLA reference set that includes; HLA-DRB1*03:01, HLA-DRB1*07:01, HLA-DRB1*15:01, HLA-DRB3*01:01, HLA-DRB3*02:02, HLA-DRB4*01:01, HLA-DRB5*01:01. The resultant epitopes with low percentile ranks were then checked for allergenicity with AlgPred server (https://webs.iiitd.edu.in/raghava/algpred/submission.html), using the support vector machine (SVM) module based on amino acid composition as the prediction approach [29]. The antigenicity and toxicity status of the non-allergenic epitopes was determined using the VaxiJen 2.0 server and ToxinPred server, respectively. Finally, interferon-gamma (IFN-$\gamma$) inducing epitopes were predicted with IFNepitope server (http://crdd.osdd.net/raghava/ifnepitope/) following the Motif and SVM hybrid approach [30]. The resultant epitopes were then inspected for overlapping.

### Population coverage

The prediction of worldwide population coverage of the selected epitopes for MHC-I and MHC-II alleles was carried out using population coverage tool of IEDB (http://tools.iedb.org/population/) [31], calculating the coverage for class I and class II separately and combined. The MHC-I alleles assessed included; HLA-B*15:01, HLA-A*30:02, HLA-A*01:01, HLA-B*40:01, HLA-B*07:02, HLA-B*51:01, HLA-A*68:02, HLA-A*02:01, HLA-A*02:06, HLA-B*08:01, HLA-A*02:03, HLA-A*33:01, HLA-A*24:02, HLA-A*23:01, HLA-B*44:03, HLA-B*44:02, HLA-A*31:01, HLA-B*53:01, HLA-A*11:01, HLA-A*68:01, HLA-A*30:01, HLA-B*57:01, HLA-A*03:01, HLA-A*26:01, HLA-B*58:01, HLA-A*32:01, HLA-B*35:0. The world coverage for these alleles was 98.55%. For MHC-II, the alleles assessed included; HLA-DRB1*07:01, HLA-DRB1*15:01, HLA-DRB3*01:01.

### B-cell epitopes prediction

The linear B-cell epitopes of all proteins under study were predicted with the antibody epitope prediction tool of IEDB (http://tools.iedb.org/bcell/) using BepiPred linear epitope prediction method 2.0 [32], Emini surface accessibility prediction method [33], and Kolaskar and Tongaonkar antigenicity method [34].

### Construction of multiepitope vaccine sequence

To ensure efficient vaccine construction and proper epitope separation, all candidate epitopes were joined together using linkers. The B-cell epitope and CTL epitopes were linked with AAY linker, and HTL epitopes were linked together and to the CTL epitopes with GPGPG linker. To facilitate future conjugation of the multi-epitope vaccine construct with a carrier protein, a cysteine residue was added at the N-terminal [35]. Furthermore, a four amino acid (EPEA) tag was added at the C-terminal for efficient purification [36]. The vaccine construct was subjected to further analysis to assess its antigenicity with VaxiJen 2.0 server, allergenicity with AlgPred server and physicochemical properties with ProtParam tool (https://web.expasy.org/protparam/) [37].

### Modeling and structure validation

The secondary structure of the novel vaccine construct was determined using PSIPRED server (http://bioinf.cs.ucl.ac.uk/psipred/) [38]. Protein modeling was carried out using threading and ab initio approaches with IntFOLD and trRosetta servers (https://www.reading.ac.uk/bioinf/IntFOLD/IntFOLD5_form.html) (https://yanglab.nankai.edu.cn/trRosetta/) [39], further protein structure analysis and model validation carried out using ProSA-web server (https://prosa.services.came.sbg.ac.at/prosa.php) [40], Ramachandran plot analysis using RAMPAGE server (http://mordred.bioc.cam.ac.uk/~rapper/rampage.php) [41] and ERRAT server (https://servicesn.mbi.ucla.edu/ERRAT/) [42].

### Molecular docking

The vaccine construct was subjected to molecular docking with Toll-like receptor -3 (TLR-3) using FRODOCK (http://frodock.chaconlab.org) and GRAMM-X simulation servers (http://vakser.compbio.ku.edu/resources/gramm/grammx/index_html), with default parameters [43].

The docked vaccine-receptor complex was then prepared for simulation using a protein-prep wizard and PyMOL software using the default settings, the molecular dynamics simulation was then carried out using the Desmond tool and Superpose1.0 server (http://superpose.wishartlab.com) for calculating the root mean square deviation (RMSD).

### Immune response simulation

The immune response to the novel multi-epitope vaccine construct was carried out using C-ImmSim server 10.1 (http://www.cbs.dtu.dk/services/C-ImmSim-10.1/) [44]**.** The simulation parameters used were, random seed: 12,345, simulation steps: 100 and simulation volume: 10 $\mu$ L. The default injection schedule with the antigen name, injection time: 0 and the injection amount: 1000.

### In silico molecular cloning

The amino acid sequence for the candidate vaccine was then subjected to reverse translation and codon optimization with JAVA Codon Adaptation Tool (Jcat) (http://www.jcat.de) [45]. The DNA sequence was then used for in silico molecular cloning with expression plasmid vector pET28::mAID from E.coli [46] using Snapgene software version 5.2.

## Results

### T-cell epitopes prediction

The initial screening of amino acid sequences of all five proteins for antigenicity, showed a score greater than the threshold value of 0.4 indicating probable antigens, these sequences were then submitted to NetCTL server to predict possible CTL epitopes, which resulted in 37 possible epitopes for S protein, out of which 14 showed no toxicity and eight positive immunogenicity score. Ultimately, the top four epitopes were selected for inclusion in the multi-epitope vaccine construct. For M protein, 10 epitopes were predicted, five epitopes showed no toxicity and only one showed a positive immunogenicity score. For E protein, three epitopes were predicted, two of which showed an antigenicity score higher than the threshold value and non-toxic, but neither showed a positive immunogenicity score, hence, not included in the vaccine construct. For N protein, nine epitopes were predicted, six showed an antigenicity score higher than the threshold value, all six predicted epitopes showed no toxicity, of which, five showed positive immunogenicity score, and only the top two were selected to be included in the construct. For the nonstructural polyprotein, on the other hand, 170 epitopes were predicted, of which 96 showed antigenicity score higher than the threshold, and the best 12 were selected based on toxigenicity and immunogenicity results (Table 1).

**Table 1:** Cytotoxic T-cell lymphocyte predicted epitopes of selected proteins based on antigenicity, toxicity, and immunogenicity

Protein	Peptide	Length	Antigenicity^a^	Toxicity^b^	Immunogenicity^c^
S	QLTPTWRVY	9 mer	1.2119	Non-toxic	0.31555
	VLPFNDGVY	9 mer	0.4642	Non-toxic	0.1815
	WTAGAAAYY	9 mer	0.6306	Non-toxic	0.15259
	CNDPFLGVY	9 mer	0.4295	Non-toxic	0.15232
M	AGDSGFAAY	9 mer	0.9095	Non-toxic	0.03981
N	LSPRWYFYY	9 mer	1.2832	Non-toxic	0.35734
	DLSPRWYFY	9 mer	1.7645	Non-toxic	0.25933
ORF1a	VSDIDITFL	9 mer	2.2906	Non-toxic	0.38916
	TLRVEAFEY	9 mer	0.4509	Non-toxic	0.34997
	HVGEIPVAY	9 mer	0.6413	Non-toxic	0.28861
	STNVTIATY	9 mer	0.7143	Non-toxic	0.25822
	LVSDIDITF	9 mer	1.7830	Non-toxic	0.2541
	NGDVVAIDY	9 mer	0.6625	Non-toxic	0.25105
	VVDYGARFY	9 mer	0.4908	Non-toxic	0.18539
	GTDPYEDFQ	9 mer	0.5315	Non-toxic	0.17381
	VTNNTFTLK	9 mer	0.7146	Non-toxic	0.16567
	ETSWQTGDF	9 mer	1.3140	Non-toxic	0.13449
	FMGRIRSVY	9 mer	0.5212	Non-toxic	0.1259
	VVVNAANVY	9 mer	0.4078	Non-toxic	0.10048

^a^Antigenicity threshold = 0.4

^b^Toxicity threshold = 0.5

^c^Rank percentile $$\le$$ 1.0

The HTL epitopes prediction with the MHC-II binding tool of IEDB and based on percentile rank less than 10, resulted in 17 epitopes for S protein, of which 12 were non-allergenic, 10 were non-toxic and a single epitope showed a positive interferon-gamma induction result. For M protein, the predicted HTL epitopes were 55, out of which 43 were non-allergenic antigenic non-toxic epitopes, and only three epitopes showed positive interferon-gamma induction results. None of the predicted HTL epitopes of N protein showed interferon-gamma positive results, therefore none were in the vaccine construct. Similarly, all HTL epitopes predicted for E protein failed to pass either the antigenicity, allergenicity, or interferon-gamma induction assessment. Out of 96 HTL predicted epitopes for ORF1a polyprotein, only six epitopes passed the antigenicity, allergenicity, toxigenicity, and interferon-gamma induction assessment. Results are shown in Table 2.

**Table 2:** Helper T-cell lymphocyte predicted epitopes of selected proteins based on antigenicity and IFN-γ response

Protein	Peptide	Length	Antigenicity^a^	IFN-gamma
S	TRFASVYAWNRKRIS	15 mer	0.4963	0.7315567
	FQTLLALHRSYLTPG	15 mer	0.5789	0.26071055
	QPYRVVVLSFELLHA	15 mer	0.9109	0.60855322
M	SRTLSYYKLGASQRV	15 mer	0.5731	0.09462399
	LVGLMWLSYFIASFR	15 mer	0.5535	0.20134879
ORF1a	VSTQEFRYMNSQGLL	15 mer	0.4972	0.97632046
	AALGVLMSNLGMPSY	15 mer	0.8521	0.11429986
	TLNGLWLDDVVYCPR	15 mer	0.4558	0.04915351
	AYESLRPDTRYVLMD	15 mer	0.5553	0.30777655
	SAGIFGADPIHSLRV	15 mer	0.5839	0.24837266
	MFTPLVPFWITIAYI	15 mer	0.6806	0.1415124

^a^Antigenicity threshold = 0.4

### B-cell epitopes prediction

**Fig. 1:**
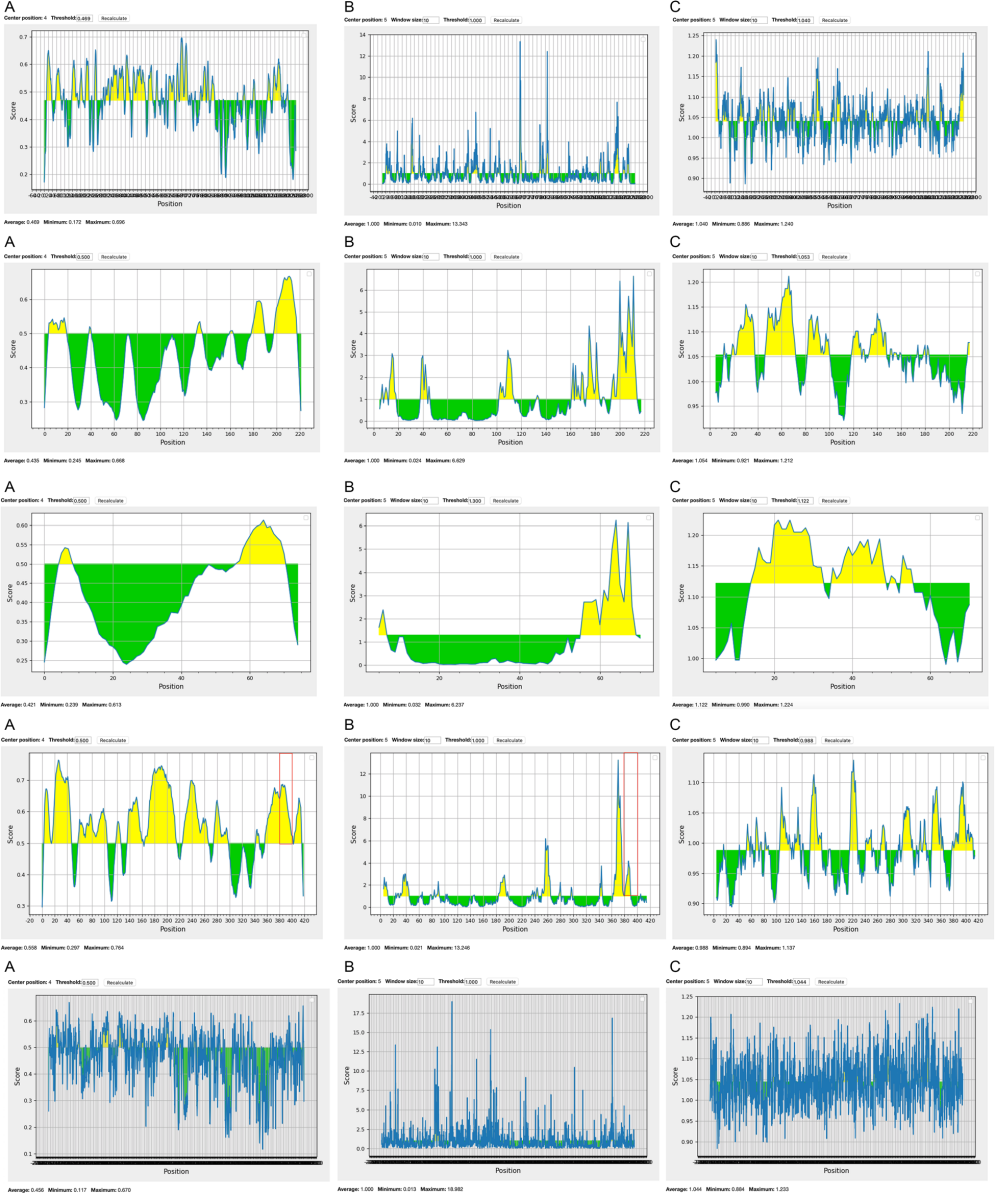
B-cell predicted epitopes of selected proteins using **A** BepiPred method; **B** Emini method; **C** Kolaskar and Tongaonkar method

The B-cell epitopes are an important part of the multi-epitope vaccine because recognition of these epitopes by B lymphocytes elicit antibody production, which is a key process in adaptive immunity. For all five proteins, linear B-cell epitopes were predicted using Bepipred Linear Epitope Prediction 2.0 method, Emini surface accessibility prediction method, and Kolaskar & Tongaonkar antigenicity method. These methods were selected because they assess properties that are important for predicting potential epitopes, such as antigenicity, surface accessibility, and flexibility. The resultant plots were then inspected for overlapping regions showing epitopes by the three methods. The only protein to show such an overlapping region was N protein with a sequence of 10 amino acids from 380–390. The results of all amino acid sequences are shown in Fig. 1.

### Population coverage

**Fig. 2:**
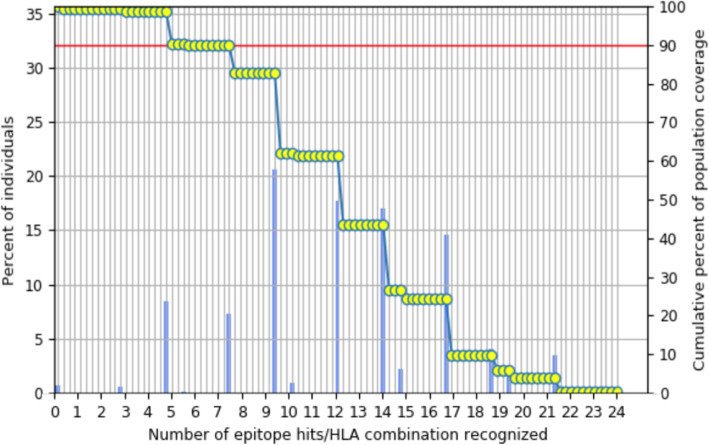
World population coverage for combined MHC-I and II alleles

The selected epitopes were then analyzed to determine the percentage of the world population coverage for MHC-I and MHC-II alleles. The coverage for these alleles was 49.02. The combine allele coverage for both MHC-I and MHC-II was found to be 99.26% which indicates a high population coverage for selected epitopes (Fig. 2).

### Multi-epitope vaccine construction

For the construction of the final vaccine construct, the most appropriate predicted epitopes were selected, this included one B-cell linear epitope from N protein, four CTL and three HTL epitopes from S protein, one CTL and two HTL epitopes from M. protein, two CTL epitopes from N protein, 12 CTL and six HTL epitopes from ORF1a. These epitopes were joined together with two types of linkers, AAY for linear B-cell and CTL epitopes, and GPGPG for HTL epitopes, with cysteine residue at the N-terminal and EPEA tag at C-terminal, this yielded the following 468 amino acid peptide chain:

CQALPQRQKKQQAAYQLTPTWRVYAAYVLPFNDGVYAAYWTAGAAAYYAAYCNDPFLGVYAAYAGDSGFAAYAAYLSPRWYFYYAAYSPDDQIGYYAAYVSDIDITFLAAYTLRVEAFEYAAYHVGEIPVAYAAYSTNVTIATYAAYLVSDIDITFAANGDVVAIDYAAYVVDYGARFYAAYGTDPYEDFQAAYVTNNTFTLKAAYETSWQTGDFAAYFMGRIRSVYAAYVVVNAANVYGPGPGTRFASVYAWNRKRISGPGPGFQTLLALHRSYLTPGGPGPGQPYRVVVLSFELLHAGPGPGSRTLSYYKLGASQRVGPGPGLVGLMWLSYFIASFRGPGPGVSTQEFRYMNSQGLLGPGPGAALGVLMSNLGMPSYGPGPGTLNGLWLDDVVYCPRGPGPGAYESLRPDTRYVLMDGPGPGSAGIFGADPIHSLRVGPGPMFTPLVPFWITIAYIGPGPGEPEA.

### Physiochemical properties of the vaccine construct

The results obtained from the ProtParam server, showed that the novel vaccine construct has a molecular weight of 50.417 KDa which is an optimum molecular weight for an antigenic protein. The theoretical isoelectric point (PI) for the construct was 5.41 indicating an acidic nature, with a total of 30 negatively charged residues and 25 positively charged residues. The estimated half-life is 1.2 h in mammalian reticulocytes in vitro, > 20 h in yeast in vivo, and > 10 h in E. coli in vivo, indicating a good construct for future cloning. The instability index was computed to be 30.79 suggesting stable protein. The aliphatic index of 75.38, which indicates a thermostable protein. The grand average of hydropathicity (GRAVY) was 0.040, a positive value close to zero means a slightly hydrophobic molecule.

### Vaccine modeling and structure analysis

**Fig. 3:**
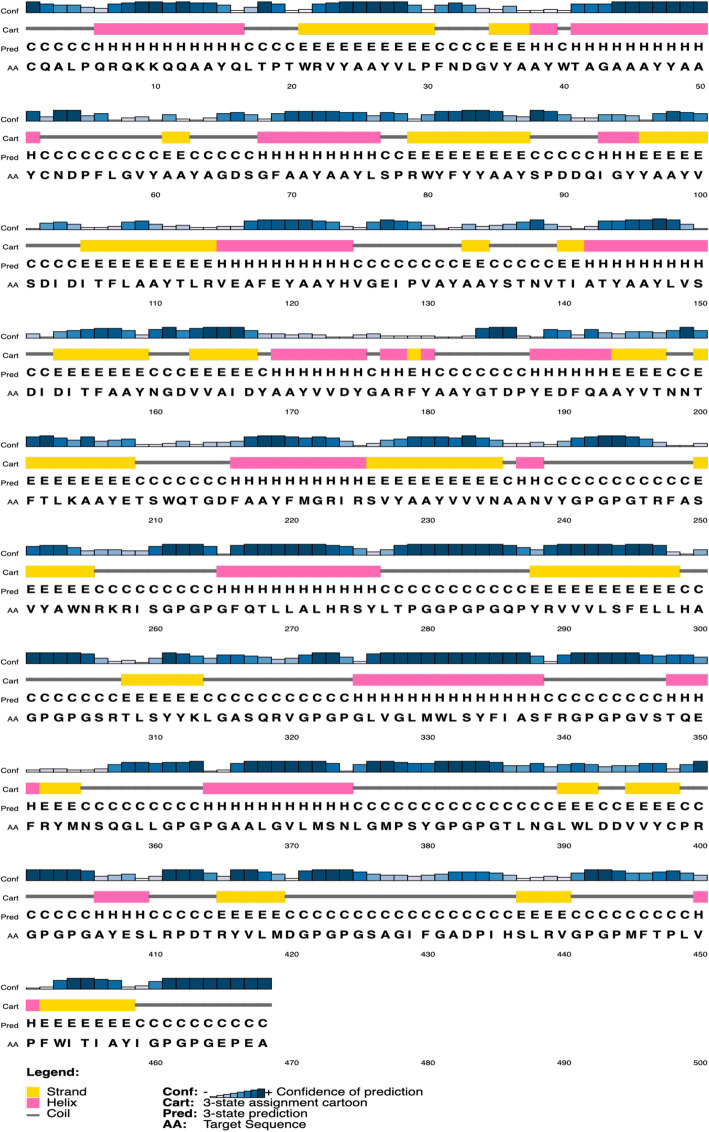
Secondary structure prediction of the novel vaccine construct

Based on the amino acid sequence of the vaccine construct, the result of the PSIPRED server revealed different secondary structures (Fig. 3). This is considered a primary step towards predicting the three-dimensional structure of the protein.

**Fig. 4:**
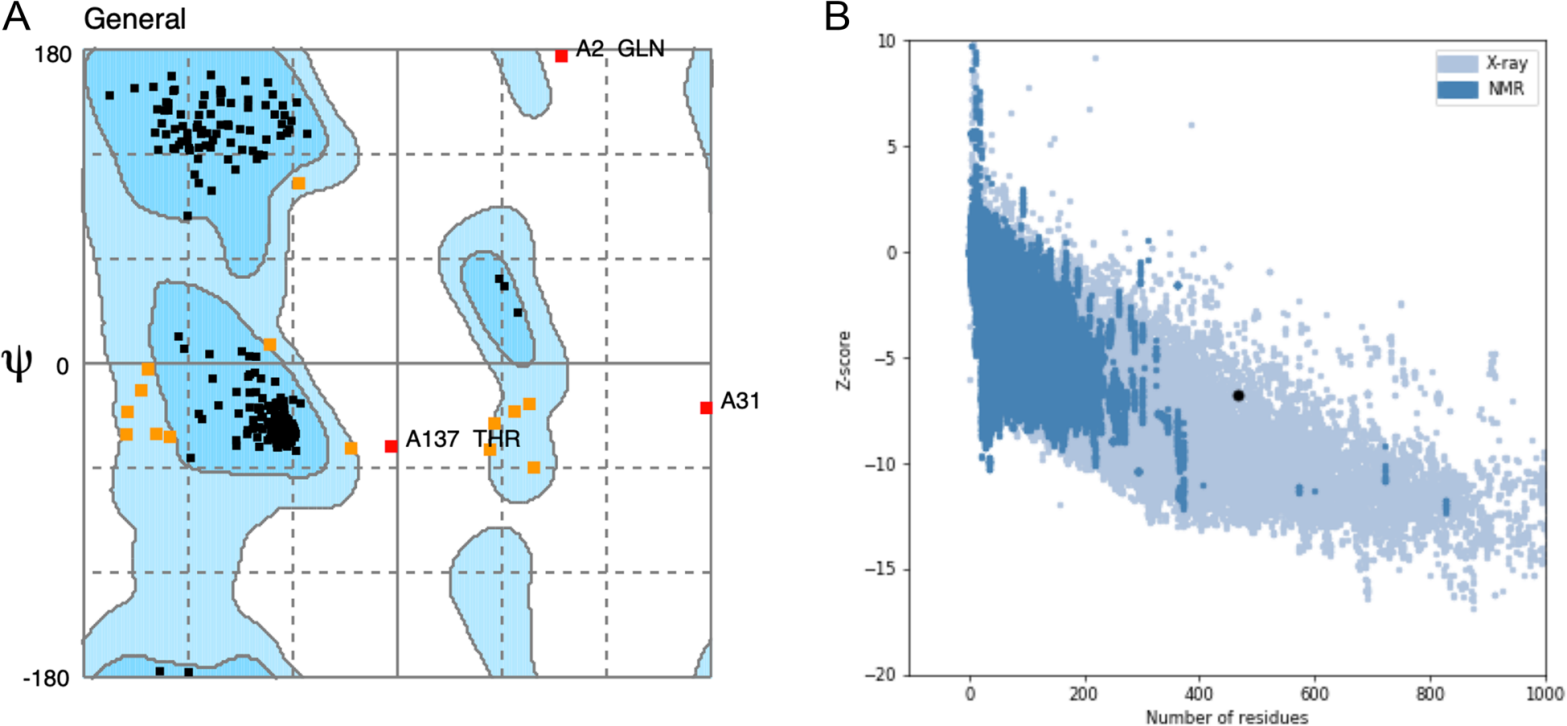
**A** Ramachandran plot showing 98% residues in the favorable region, **B** z-score determined by x-ray crystallography showing value of − 6.01

The 3D protein model was then predicted with two modeling approaches; threading model with IntFOLD server and ab initio modeling with the trRosetta server, the resultant models were then analyzed with Ramachandran plot and ProSA-web z-score based on X-ray crystallography and NMR analysis. The best-predicted model showed 98% of the residues in the favorable region in Ramachandran plot (Fig. 4A), and z-score of − 6.01, determined by x-ray crystallography (Fig. 4B).

**Fig. 5:**
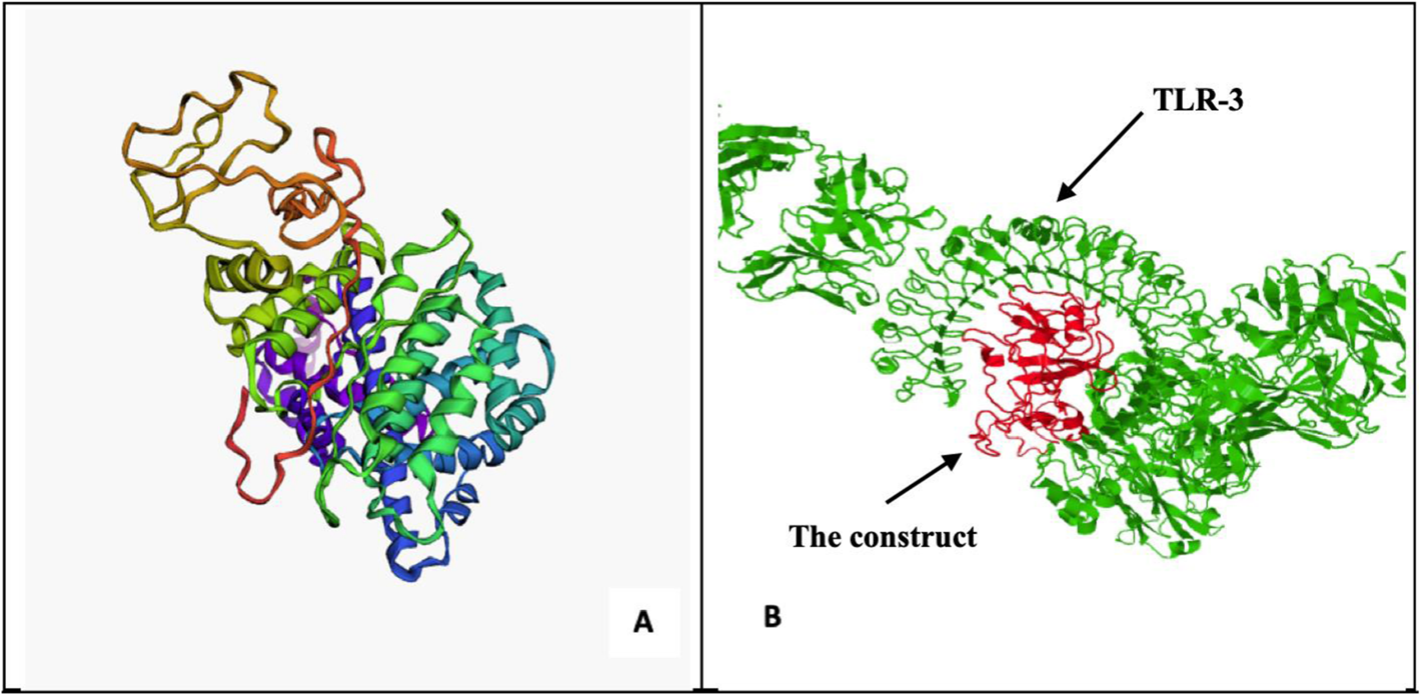
**A** 3D structure model of the candidate vaccine, **B** Docking of the vaccine construct with Toll-like receptor-3

The statistics of non-bonded interactions between different atom types and the error function value was plotted against a position of a 9-residue sliding window, calculated by comparison with statistics from highly refined structures, carried out using ERRAT server, and the calculated error value obtained was 81.928, which falls well below 91% indicating a relatively average overall quality for the selected protein model. This is can be justified by the fact that the modeling process was carried out using ab initio modeling approach (Fig. 5A).

### Molecular docking and dynamics

The final vaccine construct was docked with Toll-like receptor 3 (PDB ID: 1ziw) using the FRODOCK server. The value of the root mean square deviation was 3.78 which suggests a relatively poor binding pose at the site of the receptor and vaccine binding Fig. 5B.

### Immune response simulation

Measuring the immune response is a pivotal step for vaccine designing and this is contingent on a number of algorithms that make use of mathematical models to illustrate the fine details of the immunological process. In the present study, the C-ImmSim server was used to simulate immune response with the candidate vaccine construct. Simulation with this tool focuses on B-cell epitope binding, class I and II HLA epitope binding, and the binding of the T-cell receptor to HLA-peptide complexes [47].

**Fig. 6:**
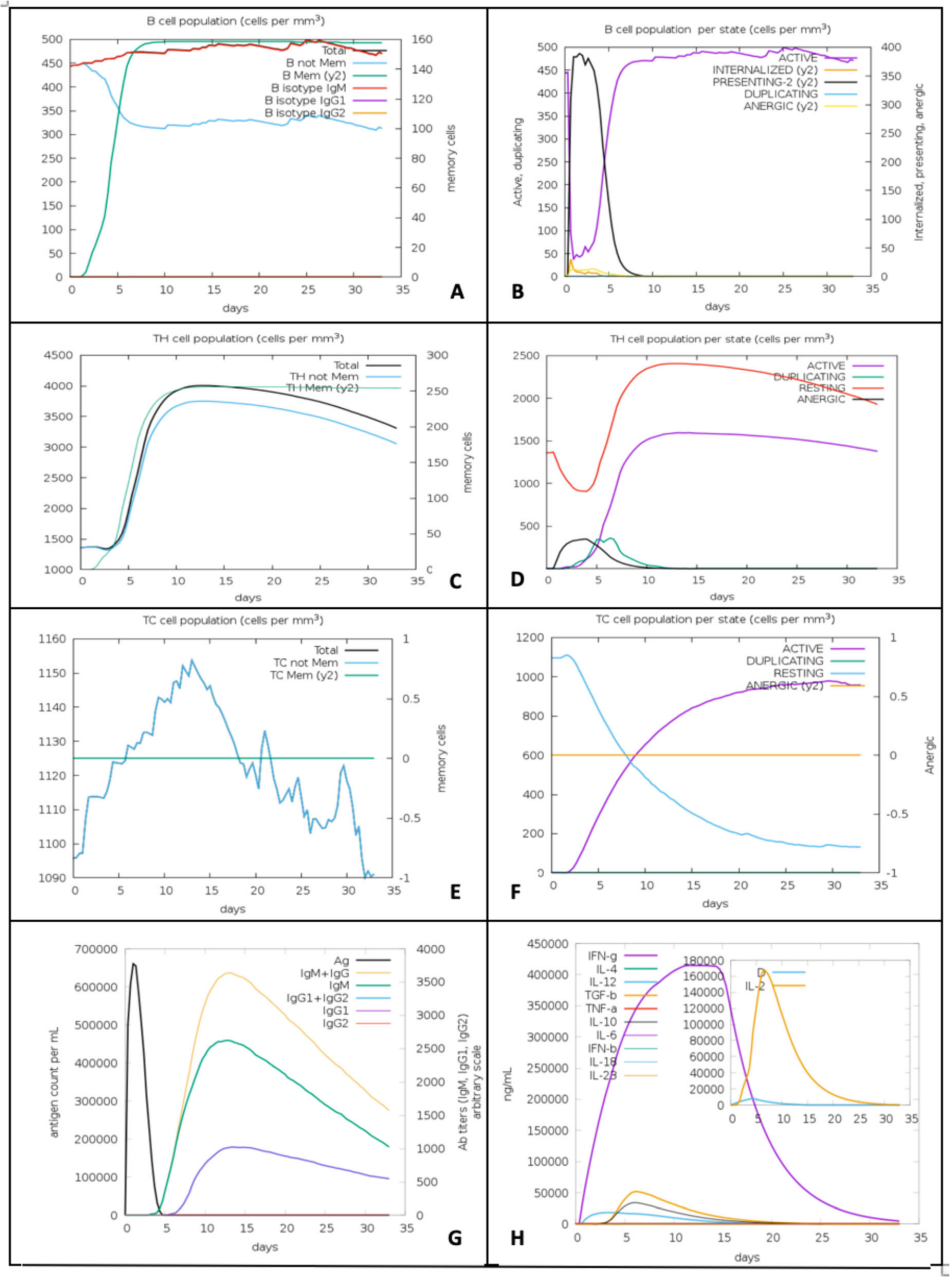
Different immune responses simulation of the vaccine construct using C-ImmSim. **A** B Cell population (cells/mm^3^), **B** B Cell population per state (cells/mm^3^), **C** TH Cell population (cells/mm^3^), **D** TH Cell population (cells/mm^3^), **E** TC cell population (cells/mm^3^), **F** TC cell population per state (cell/mm^3^), **G** Concentration of immunoglobulins & immunocomplexes, **H** Concentration of cytokines & interleukins

The simulation results showed an increased and sustained level of B- memory and active cells, and a high level of IgM, which represents the primary response against the antigen and this suggests effective humoral response (Fig. 6A, B). T helper cell population showed very promising results, as the levels of memory helper cells and active T helper cells remained high for the entire period of simulation, suggesting prolonged humoral and cell-mediated immune response (Fig. 6C, D). The results of the T cytotoxic cell population showed a steady level of the memory cells, while the active cell population showed an increased level throughout the stimulation period (Fig. 6E, F). The result of different immunoglobulin isotopes showed high level in the first two weeks followed by a gradual decline, similar result was shown by interferon-gamma level. This can be viewed as a positive point, since, the first two weeks are considered detrimental for the course and outcome of the disease (Fig. 6G, H) [48].

### In silico molecular cloning

**Fig. 7:**
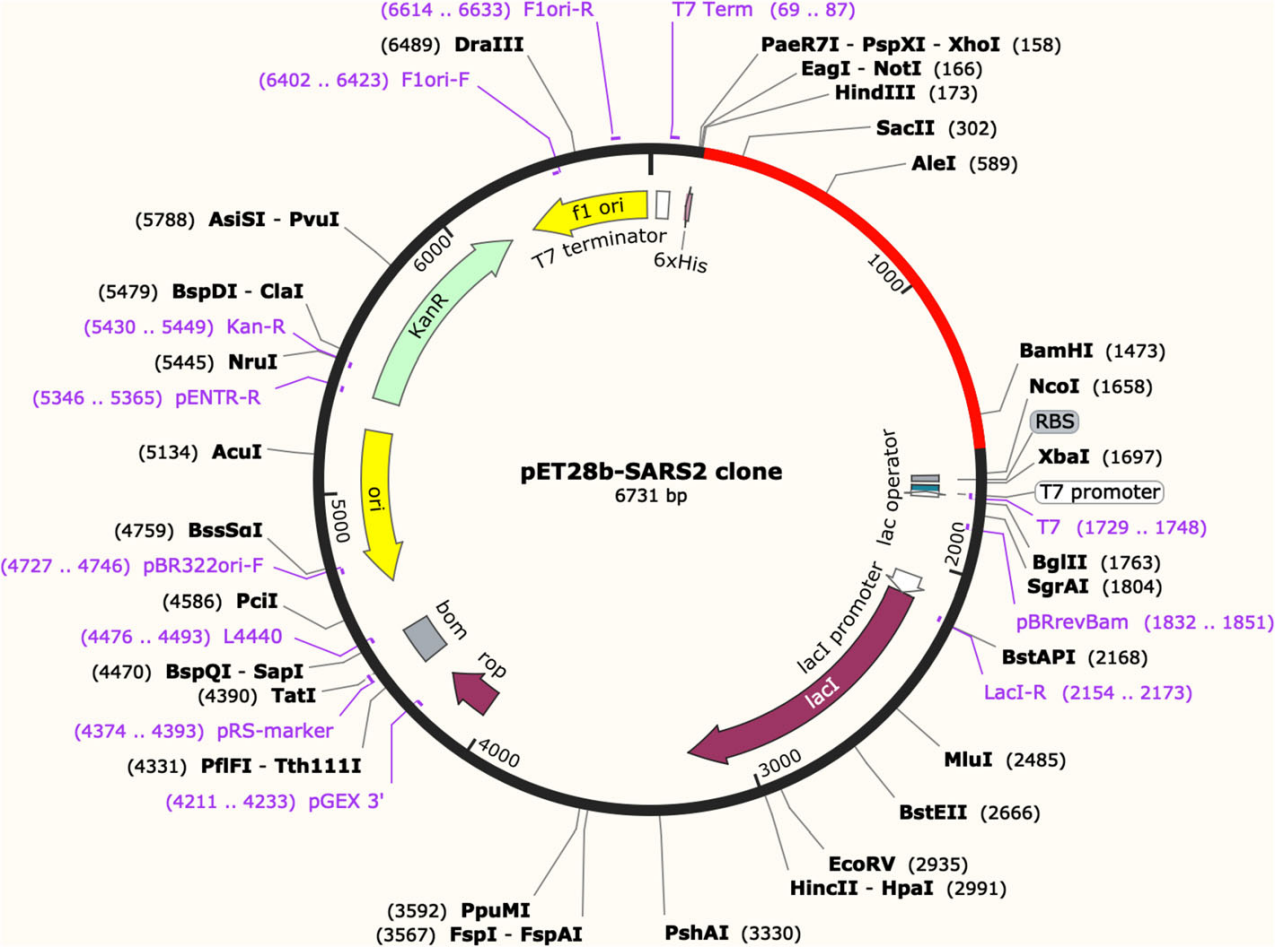
In silico cloning of vaccine construct using pET28b plasmid

The DNA sequence produced by Jcat showed a GC content of 56% and a codon adaptation index of 1.0, which indicate a stable DNA sequence and a high level of protein expression (Fig. 7).

## Discussion

The current COVID-19 pandemic associated with SARS-CoV-2 infection is the third coronavirus outbreak in the last 20 years besides the severe acute respiratory syndrome (SARS) and the Middle East respiratory syndrome (MERS). SARS-CoV-2 shows relatively higher transmissibility as compared to other emerging viruses such as H7N9 and MERS-CoV [49, 50]. This entails the imperative search for effective vaccine and treatment in addition to the protective and social distancing measures to contain and control the disease. The immunoinformatics approach provides a promising tool for designing and exploring potential vaccines against bacterial, parasitic, and viral diseases [51]. In this study, a multi-epitope vaccine was constructed using the virus structural proteins and the largest non-structural polyprotein [52]. These proteins were selected based on suggestions from previous studies [53, 54]. Unlike the single subunit vaccine, the multi-epitope vaccine is believed to induce a better and more protective immune response [55].

A number of previously conducted studies used similar approach to construct multi-epitope vaccines, however, unlike our present study, these previous attempts used either the structural proteins alone for constructing the vaccine [56, 57] or the spike protein and one non-structural protein [58], or entire set of viral proteins.

In the present study antigenic, non-allergenic, and non-toxic epitopes were identified and used for the construction of the final candidate vaccine. All five proteins were studied for potential epitopes, however, none of the peptides from the envelope protein (E) was eligible for the selection in the final vaccine construct, due to either lack of antigenicity or the allergenicity and toxicity of these peptides, this can also be attributed to the small size of the protein. For the final vaccine construct, CTL, HTL, line B-cell epitopes were linked together using AAY, and GPGPG linkers which provide proper proteasomes cleavage sites for different immune cells [59] which will ultimately enhance the antigen presentation process by binding transporters associated with antigen processing (TAP) [60]. Furthermore, linking of CTL epitopes from different proteins together forms epitopes on a string which is believed to enhance the immunogenicity of CTL epitopes [61]. To the N-terminal of the vaccine construct a cysteine residue was added to facilitate the binding of this vaccine to protein carrier [35], and to the C-terminal, a small peptide of four amino acids EPEA was added to enable downstream purification process [36]. The candidate vaccine construct consists of 486 amino acids, which is an ideal vaccine length, since larger proteins are presented by dendritic cells leading to stronger T-cell immune response [60], while extremely short peptides may induce tolerance and energy by directly binding MHC molecules of non-professional antigen-presenting cells [62]. Determination of the secondary structure of the protein is a pivotal step towards the prediction of its three-dimensional structure, therefore, the secondary structure of the candidate vaccine was determined using PSIPRED server, followed by structure refinement, and protein modeling. Two approaches were used for modeling the protein, threading approach, and ab initio approach, the best resultant model was selected based on the Ramachandran plot and z-score analyses. The docking of the vaccine and TLR-3 showed a possible hydrophilic interaction [63], this interaction indicates a possible recognition of the vaccine by APC specific receptor, which in turn promotes the immune response [64]. The results of immune response simulation showed very promising results, with a sustained response for the cells involved in the humoral and cell-mediated immunity against SARS-CoV-2. Even though most of the currently in-use vaccines are showing high degrees of effectiveness and safety, the potential future risks cannot be overlooked. Three of these vaccines elicit the immune response against a single viral protein, namely, S protein, however, the recent emergence of number of new variants has cast doubt on the effectiveness of the currently used vaccines, with reports claim that the E484K mutation found in the South African (B.1.351) and Brazilian (B.1.128) variants has a negative impact on the longevity of the neutralizing antibodies and, possibly, the vaccine effectiveness [65]. Other studies reported reduced protection of BNT162b2 vaccine against B.1.351 variant [66], and lack of protection of ChAdOx1 nCoV-19 vaccine against the same variant [67]. Many of the recently identified mutations occur in the viral spike gene conferring antibody neutralization resistance [68], and the accumulation of such mutations is believed to ultimately render the current vaccines directed against the viral spike protein ineffective [69]. The proposed multi-epitope vaccine is designed using several structural and non-structural proteins which makes it an appropriate alternative.

The mRNA vaccines also require certain environmental conditions for preservation of the highly unstable nucleic acid. On the other hand, the inactivated vaccines, though elicit a comprehensive immune response against larger number of viral proteins, there are inherent problems associated with viral inactivation process, and the time-consuming production process.

The conventional methods of vaccine development are very costly and time-consuming, alternatively, the immunoinformatics approach has attracted the attention as an ideal method for designing less-expensive, rapid, efficient, multi-epitope vaccines. However, experimental validation is of utmost importance to ensure the safety and efficacy of the resultant vaccine, it is also beyond the scope of this study to explore any possible pathogenic priming or autoimmune disease induction of the proposed vaccine.

## Conclusion

The highly contagious nature of SARS-CoV-2 left the entire world population with no option but to wait for the production of a safe and protective vaccine to break the chain of infection and tackle the spread of this pandemic. It is rather impractical to rely on the conventional methods for producing such a vaccine due to a number of limiting factors. This study is an attempt to design an efficient multi-epitope chimeric subunit vaccine that is capable of mounting a strong immune response by induction of both humoral and cellular mediated immunity, with the help of a large number of immunoinformatics tools. The vaccine construct effectively fulfilled the requirements for characteristics such as antigenicity, allergenicity, immunogenicity, physiochemical properties, eliciting the immune response in a simulation model. It is concluded that this novel construct represents a promising candidate for an efficient protective vaccine against SARS-CoV-2.

## Data Availability

Not applicable.

## Abbreviations

ACE-2: Angiotensin converting enzyme 2
ARDS: Acute respiratory distress syndrome
Covid-19: Coronavirus disease-19
CTL: Cytotoxic T lymphocyte
DNA: Deoxyribonucleic acid
E. protein: Envelope protein
GRAVY: Grand average of hydropathicity
HLA: Human leukocyte antigen
HTL: Helper T lymphocyte
ICTV: International committee on Taxonomy of Viruses
IEDB: Immune epitope database
IFN-$\gamma$: Interferon gamma
Kb: Kelo base
KDa: Kelo Dalton
M. protein: Membrane protein
MERS-CoV: Middle East Respiratory Syndrome coronavirus
MHC: Major histocompatibility complex
mRNA: Messenger RNA
N. protein: Nucleocapsid protein
NMR: Nuclear magnetic resonance
ORF: Open reading frame
PDB: Protein database
PI: Isoelectric point
PP1a: Polyproteins 1a
RMSD: Root mean square deviation
RNA: Ribonucleic acid
S. protein: Spike protein
SARS-CoV-2: Sever acute respiratory syndrome coronavirus-2
SVM: Support vector machine
TLR: Toll-like receptor
WHO: World Health Organization
